# Methods and associations of suicidality in Kenyan high school students: clinical and public health implications

**DOI:** 10.1192/bjo.2024.56

**Published:** 2024-05-13

**Authors:** David M. Ndetei, Danuta Wasserman, Victoria Mutiso, Jenelle R. Shanley, Christine Musyimi, Pascalyne Nyamai, Timothy Munyua, Monica H. Swahn, Tom L. Osborn, Natalie E. Johnson, Peter Memiah, Kamaldeep Bhui, Sonja Gilbert, John R. Weisz, Afzal Javed, Andre Sourander

**Affiliations:** Africa Mental Health Research and Training Foundation, Nairobi, Kenya; Department of Psychiatry, University of Nairobi, Nairobi, Kenya; and World Psychiatric Association Collaborating Centre for Research and Training, Nairobi, Kenya; Karolinska Institute, Stockholm, Sweden; Africa Mental Health Research and Training Foundation, Nairobi, Kenya; and World Psychiatric Association Collaborating Centre for Research and Training, Nairobi, Kenya; Pacific University, Hillsboro, USA; Department of Health Promotion and Physical Education, Wellstar College of Health and Human Services, Kennesaw State University, Kennesaw, USA; Shamiri Institute, Nairobi, Kenya; Shamiri Institute, Nairobi, Kenya; Division of Clinical Epidemiology, Department of Clinical Research, University Hospital Basel, Basel, Switzerland; and University of Basel, Basel, Switzerland; University of Maryland, Baltimore, USA; Department of Psychiatry, Nuffield Department of Primary Care Health Sciences, Wadham College, University of Oxford, UK; National Institute for Health Research (NIHR) Oxford Health Biomedical Research Centre; Oxford, UK; and World Psychiatric Association Collaborating Centre, Oxford, UK; Research Centre for Child Psychiatry, Department of Clinical Medicine, Faculty of Medicine, University of Turku, Turku, Finland; and INVEST Child Psychiatry, INVEST Research Flagship Center, Department of Clinical Medicine, Faculty of Medicine, University of Turku, Turku, Finland; Department of Psychology, Harvard University, Cambridge, MA, USA; World Psychiatric Association, Geneva, Switzerland; Research Centre for Child Psychiatry, Department of Clinical Medicine, Faculty of Medicine, University of Turku, Turku, Finland; INVEST Child Psychiatry, INVEST Research Flagship Center, Department of Clinical Medicine, Faculty of Medicine, University of Turku, Turku, Finland; and Department of Child Psychiatry, Turku University Hospital, Turku, Finland

**Keywords:** Suicide methods, suicide thoughts, suicide plans, suicide attempts, prevalence

## Abstract

**Background:**

Most evidence on suicidal thoughts, plans and attempts comes from Western countries; prevalence rates may differ in other parts of the world.

**Aims:**

This study determined the prevalence of suicidal thoughts, plans and attempts in high school students in three different regional settings in Kenya.

**Method:**

This was a cross-sectional study of 2652 high school students. We asked structured questions to determine the prevalence of various types of suicidality, the methods planned or effected, and participants’ gender, age and form (grade level). We provided descriptive statistics, testing significant differences by chi-squared and Fisher's exact tests, and used logistic regression to identify relationships among different variables and their associations with suicidality.

**Results:**

The prevalence rates of suicidal thoughts, plans and attempts were 26.8, 14.9 and 15.7%, respectively. These rates are higher than those reported for Western countries. Some 6.7% of suicide attempts were not associated with plans. The most common method used in suicide attempts was drinking chemicals/poison (18.8%). Rates of suicidal thoughts and plans were higher for older students and students in urban rather than rural locations, and attempts were associated with female gender and higher grade level – especially the final year of high school, when exam performance affects future education and career prospects.

**Conclusion:**

Suicidal thoughts, plans and attempts are prevalent in Kenyan high school students. There is a need for future studies to determine the different starting points to suicidal attempts, particularly for the significant number whose attempts are not preceded by thoughts and plans.

## Prevalence of suicide thoughts, plans and attempts

The estimated prevalence of suicidal thoughts, plans and attempts varies widely across different studies and countries.^1–10^ Among young people, the prevalence of suicidal thoughts has been reported to range from 10.7% in a cross-sectional epidemiological study in Germany^7^ to 17% in a prospective cohort study in Norway^4^ and 17.9% in a study in Turkey,^5^ whereas the prevalence of suicide attempts ranges from 3.6 to 10.3%.^6,7^ In the USA, the prevalence of suicidal thoughts ranged from 4.0% in the north-east and south to 4.8% in the west, and from 3.3% in New Jersey to 6.9% in Utah in the National Survey on Drug Use and Health covering the period between 2015 and 2019.^11^

Differences in prevalence have been observed in African studies, with lifetime prevalence rates of suicidal thoughts, plans and attempts of 58.3, 37.3 and 4.4%, respectively, reported in an Ethiopian cross-sectional study.^10^ A cross-sectional study in Ghana^9^ found prevalence rates of 15.4, 6.6 and 2.3%, respectively. The study in Ghana focused on nursing students specialising in midwifery; thus, it provided a broader perspective on the prevalence of suicidality among various populations. In addition, a study on suicidal ideation among youth in Kampala, Uganda, reported a prevalence of 23.53%.^8^

A study of suicidal ideation among Kenyan students aged 15+ years found a prevalence of 22.6%.^1^ In an out-patient psychiatric clinic in a youth centre of a referral hospital in Kenya, 82% of youth showed suicidal behaviours (plans and attempts); those aged 16–18 years were more likely to show suicidal behaviour compared with those aged 19–22 years.^2^

## Methods in suicidality

According to the World Health Organization (WHO), hanging, ingestion of pesticides and use of firearms are the most common methods of suicide globally.^12^ Among adolescents, a meta-analytic review of worldwide suicide rates reported that the most common suicide methods were hanging and jumping from a height or in front of a moving object.^13^ A study in Western Kenya^14^ found the most commonly reported method of suicide to be self-poisoning. Research has shown that suicidality methods tend to differ, with less lethal methods reported in attempters and highly lethal methods for completers.^15^ A study in Korea found drug poisoning (which they classified as a less lethal method) to be the most frequent method used by suicide attempters, whereas hanging (classified as highly lethal) was the most common method among suicide completers.^15^

## Suicidality associations

There is evidence of an association of suicide attempts with thoughts and plans. In the German study mentioned previously,^7^ 47.0% of participants who had lifetime suicidal ideation reported a suicide plan, and 23.9% reported a suicide attempt. Suicidal plans or attempts occurred mainly in the year of suicidal ideation, with 74.9% of plans and 71.2% of attempts associated with ideation and 85% of attempts associated with plans.^7^

Prevention of suicide requires good evidence on both the prevalence and probable hot spots, as well as suicidal behaviours, to inform the development of appropriate interventions for communities and for health and social systems.^16^ There is a gap in such knowledge in the Kenyan context. Therefore, in this study, we sought to determine the levels of suicidal thoughts, plans, and attempts; suicidality patterns; and sociodemographic associations of suicidality in high school students in three different regional settings in Kenya.

The specific objectives were to determine the following:

- the prevalence of suicidal thoughts, plans and attempts;
- methods contemplated in suicidal plans and employed in suicidal attempts;
- patterns of suicidal thoughts and plans to suicidal attempts;
- sociodemographic associations of suicidal thoughts, plans and attempts.

## Method

### Study setting

We undertook a cross-sectional study in ten secondary schools. The schools were sampled from three regions of 47 (otherwise referred to as counties) in Kenya, which are geographical units created by the 2010 Constitution of Kenya. Our decision to focus on these three regions was primarily influenced by an ongoing project we had in those counties. It made logistical sense to extend our efforts into these areas where we already had established connections, networks and perhaps even existing data that could inform our work. The sampling sought to ensure we recruited a demographically representative sample that reflected the diversity of adolescents within Kenyan secondary schools. The institutions also represented all four levels of government-funded schools (national, extra-county, county and sub-county – the main differences between these types of school in Kenya are their ownership, management and the level of academic performance required for admission) as well as urban and rural school locations. Ten schools were selected as follows: one national, one extra-county, five county and three subcounty. Three schools were day schools, five were boarding, and two had both day and boarding facilities. All the schools had a guidance and counselling department supported by trained teachers. This is in line with a policy directive, where the government has committed resources towards school counselling programmes to assist students to deal with behavioural and psychological challenges that affect their academic and social relations.^17^ Our deliberate choice to prioritise public schools over private ones was informed by a range of factors, including resource limitations and the potential psychological impact on students. Public schools often face more significant resource constraints compared with their private counterparts, and this can have profound effects on students’ well-being. These limitations can manifest in various ways, including inadequate mental health support systems, fewer extracurricular opportunities and larger class sizes. These in turn can contribute to increased levels of stress, anxiety and even suicidal ideation among students in public schools.

### Sampling procedure

Sampling was multi-stage, with a broad stratification rationale applied to first select the regions, then the secondary schools that represented each of the four levels of government-funded schools within the three regions selected. In each of the schools, students were randomly divided into small groups assembled for the study. Each group comprised 10–15 students, with one research assistant in charge of each group. The questionnaire was thereafter self-administered to the students using paper and pencil.

### Data collection to achieve objectives 1, 2 and 3

We asked structured questions to determine the prevalence and methods of suicidal behaviour. Five questions were asked, as follows. (a) ‘Have you thought seriously about ending your life?’ (‘No, I have not,’ ‘Yes, once,’ ‘Yes, more than once’). For this analysis, the response options were dichotomised into ‘No’ and ‘Yes’. (b) ‘Have you tried ending your life?’ (‘No, I have not,’ ‘Yes, once,’ ‘Yes, more than once’). For this analysis, the response options were dichotomised into ‘No’ ‘and ‘Yes’. (c) ‘If yes to question 1 above, did you think of a possible way to end your life?’ (Yes/No). (d) ‘If yes to question 3 above, how?’ (list the methods). (e) ‘If yes to question 2 above, what methods did you use?’ (list them).

### To achieve objective 4

Demographic data were assessed using three self-reported questions: (a) gender (male/female/other); (b) age (in years)’; and (c) ‘In what form (high-school grade) are you?’ (1, 2, 3 or 4).

### Statistical analysis

Data analysis was performed with SPSS version 25 (Armonk, NY: IBM Corp) for Microsoft Windows. Descriptive summary statistics in the form of frequency, percentage, mean of the age and standard deviation were generated to examine the variables. χ^2^ and Fisher's exact tests were used where appropriate to determine significance. Univariate and multivariate logistic regression was performed to determine: (a) which socio-demographic variables were associated with suicidality; (b) which suicide plan methods were associated with suicide attempts; and (c) associations between sociodemographic variables and the various suicidality patterns.

### Ethics

The authors assert that all procedures contributing to this work complied with the ethical standards of the relevant national and institutional committees on human experimentation and with the Helsinki Declaration of 1975, as revised in 2008. All procedures involving human subjects were approved by Kenyatta University Ethics Review Committee (protocol number: PKU/2456/E1587). A research license was granted from the National Commission for Science, Technology, and Innovation (licence number: NACOSTI/P/22/17173). Permission to conduct this research was sought from institutional heads. Informed written consent was obtained from those students over 18 years and assent from those under 18 years. In addition, written consent was obtained from parents/guardians of participants under 18 years via school officials. We assumed there would be students who had suicidal thoughts and/or plans or had attempted suicide. As this was an anonymous survey, we had no way of identifying the students with any of these. In mitigation, we advised the students that in the event they had any of these, they could discuss them with trusted friends, teachers, school counsellors and family members if they felt comfortable with them and gave them the option of a helpline at the Africa Mental Health Research and Training Foundation (AMHRTF).

## Results

### Sociodemographic characteristics

Of the 2652 participants, 66.6% were male, 33.2% were female and 0.2% were ‘other’. The mean age was 16.3 years, with a standard deviation of ±1.38. The age of the participants ranged from 14.75 to 17.51 years, where average ages were as follows: form 1, 14–15 years; form 2, 15–16 years; form 3, 16–17 years; and form 4, 17–18 years. The fewest respondents (13.6%) were in form 4 (the final class in high school). More of the schools were rural (61.3%) than urban (38.7%). Table 1 provides a summary of the sociodemographic characteristics.

**Table 1:** Sociodemographic characteristics of participants

Variable	Category	*n* (%)
Gender	Female	862 (33.2)
	Male	1728 (66.6)
	Other	6 (0.2)
Age, years	Mean (s.d.)	16.13 (1.38)
	Median (interquartile range)	16.00 (15.00, 17.00)
	Range	13.00, 23.00
Form	1	869 (33.5)
	2	646 (24.9)
	3	729 (28.1)
	4	352 (13.6)
Location of school	Rural	1627 (61.3)
	Urban	1025 (38.7)

### Prevalence of suicidality and its patterns

The response rate for the suicidality questions (suicidal thoughts, suicide plans and suicide attempts) was 95.6% (2534/2652). The responses for various aspects of suicidality could be grouped into the following four categories (percentages/*n*):

- suicidal attempts (regardless of suicidal ideas or plans): 15.7% (397/2534);
- suicidal plans but no attempt (regardless of suicidal ideation) 14.9% (377/2534);
- suicidal ideations (regardless of plans and attempts) 26.8% (680/2534);
- no suicidality (no ideations, no plans, no attempts) 42.6% (1080/2534).

The associations and co-occurrences among suicidal thoughts, plans and attempts can be summarised as follows.

#### Suicidal thoughts

Of the 680 respondents who had had suicidal thoughts: (a) 55.4% (377/680) had made suicide plans, whereas 44.6% (303/680) had not; (b) 51.3% (349/680) had attempted suicide; (c) 36.0% (245/680) had attempted once and 15.3% (104/680) more than once.

#### Suicidal plans

Of the 377 respondents who had made suicide plans: (a) 67.1% (253/377) had attempted suicide; (b) 44.8% (169/377) had attempted suicide once and 22.3% (84/377) more than once.

#### No suicidal plans

Of the respondents who had not made suicide plans but had had suicidal thoughts: (a) 31.7% (96/303) had attempted suicide; and (b) 25.1% (76/303) had attempted suicide once and 6.6% (20/303) more than once. Of the respondents who had no suicide plans or suicidal thoughts: (a) 6.7% (144/2157) had attempted suicide; (b) 5.6% (120/2157) had attempted suicide once and 1.1% (24/2157) more than once.

#### No suicidal thoughts

**Fig. 1:**
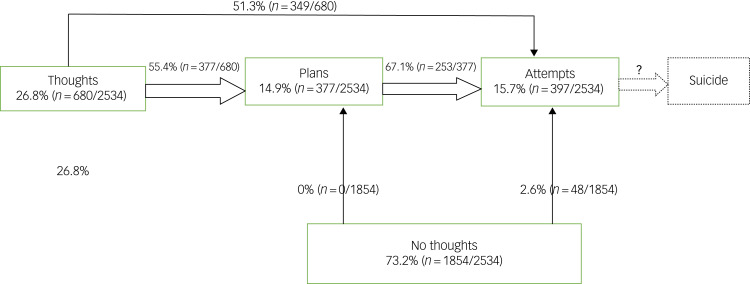
Different starting points towards suicide attempts, demonstrating that: there was a decrease in overall prevalence of thoughts, plans and attempt; it is possible to move from suicidal thoughts to attempts, bypassing plans; attempts may occur without thoughts; and attempts may occur without plans. We did not have information on completed suicide.

A total of 73.2% (1854/2534) did not have suicidal thoughts. Of those who did not have suicidal thoughts, 2.6% (48/1854) had attempted suicide – 2.4% (44/1854) once and 0.2% (4/1854) more than once. See Table 2 and Fig. 1 for a summary of suicidality prevalence and its patterns

**Table 2:** Patterns of suicidality among 2534 (of a sample of *N* = 2652) participants who reported suicidality

Prevalence of suicidality	
Overall prevalence (*N* = 2534)	
Suicidal thoughts	26.8% (680/2534)
Suicidal plans	14.9% (377/2534)
Suicidal attempts	15.7% (397/2534)
Prevalence of combinations of suicidality (*N* = 2534)	
Thoughts + plans + attempts	9.9% (253/2534)
Thoughts + no plans + attempts	3.8% (96/2534)
No thoughts + no plans + attempts	1.9% (48/2534)
More suicidal attempts than plans	
No suicidal thoughts, no plans, but suicidal attempts	2.6% (48/1854)
Suicidal thoughts, no suicidal plans but suicidal attempts	31.7% (96/303)
Subtotal of suicidal attempts with no plans	6.7% (144/2157)
Tracing through progression from suicidal thoughts to plans and to attempts	
Suicidal thoughts	26.8% (680/2534)
Suicidal plans – those with suicidal thoughts who developed suicidal plans	55.4% (377/680)
Suicidal attempts – those with suicidal plans who made suicidal attempts	67.1% (253/377)
Breakdown of suicidal attempts (*n* = 397)	
Total number following through from suicidal thoughts, plans and attempted suicide	253 (63.7%)
Thoughts, no suicidal plans + suicidal attempts	96 (24.2%)
No suicidal thoughts, no suicidal plans + suicidal attempts	48 (12.1%)

### Methods in suicidal plans and attempts

The methods contemplated in suicidal plans were the same as those used in suicide attempts. The most common method used in suicide attempts was drinking chemicals/poison (18.8%), whereas the least used method was ‘suicide by cop’ (0.3%). On the other hand, hanging and drinking chemicals/poisons were the most common methods contemplated in suicidal plans. There were no significant associations between gender and suicide attempt methods.

**Fig. 2:**
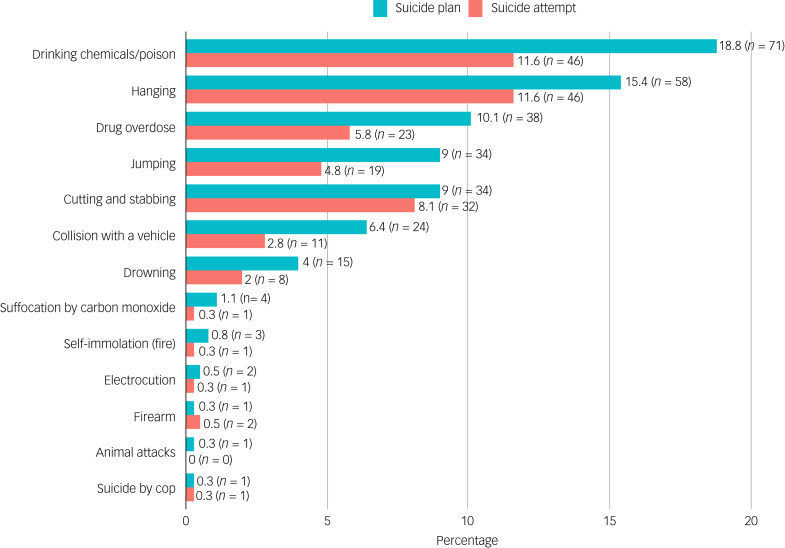
Methods reported in suicidal plans and attempts (suicide plan, *N* = 377; suicide attempt, *N* = 397). The number at the end of each bar indicates the prevalence (as a percentage) of the specific suicide method plan.

Significant gender differences were observed between suicide plan methods for drowning, drug overdose and suffocation by carbon monoxide. Males had a significantly (*P* < 0.05) higher proportion of drowning suicide plans compared with females. (6.2% *v.* 0%), and females had a significantly higher proportion of drug overdose suicide plans compared with males. (14.8% *v.* 7.4%). Males and females had significantly lower proportions of suffocation by carbon monoxide suicide plans compared with ‘other’ gender. There were no significant differences between rural and urban locations in proportions of methods reported in suicide plans and suicide attempts. See Fig. 2 for a summary of the methods contemplated in suicidal plans and used in attempted suicides, and Table 3 for details of sociodemographic differences in methods involved in suicide plans and attempts.

**Table 3:** Suicide methods stratified by gender and location

Variable	Category	Total (*N* = 2596)	Gender			*P*-value	Total (*N* = 2652)	Location		*P*-value
			Female	Male	Other			Rural	Urban	
Suicide plan – drinking chemicals/poison	No	304 (81.3%)	107 (83.6%)^a^	194 (79.8%)^a^	3 (100.0%)^a^	0.640	306 (81.2%)	174 (83.3%)	132 (78.6%)	0.248 (χ^2^)
	Yes	70 (18.7%)	21 (16.4%)^a^	49 (20.2%)^a^	0 (0%)^a^		71 (18.8%)	35 (16.7%)	36 (21.4%)	
Suicide plan – jumping	No	340 (90.9%)	120 (93.8%)^a^	217 (89.3%)^a^	3 (100.0%)^a^	0.390	343 (91.0%)	193 (92.3%)	150 (89.3%)	0.303 (χ^2^)
	Yes	34 (9.1%)	8 (6.3%)^a^	26 (10.7%)^a^	0 (0%)^a^		34 (9.0%)	16 (7.7%)	18 (10.7%)	
Suicide plan – drowning	No	359 (96.0%)	128 (100.0%)^a^	228 (93.8%)^b^	3 (100.0%)^a, b^	0.007	362 (96.0%)	198 (94.7%)	164 (97.6%)	0.155 (χ^2^)
	Yes	15 (4.01%)	0 (0%)^a^	15 (6.2%)^b^	0 (0%)^a, b^		15 (4.0%)	11 (5.3%)	4 (2.4%)	
Suicide plan – cutting and stabbing	No	340 (90.9%)	116 (90.6%)^a^	221 (90.9%)^a^	3 (100.0%)^a^	>0.999	343 (91.0%)	194 (92.8%)	149 (88.7%)	0.164 (χ^2^)
	Yes	34 (9.1%)	12 (9.4%)^a^	22 (9.1%)^a^	0 (0%)^a^		34 (9.0%)	15 (7.2%)	19 (11.3%)	
Suicide plan – hanging	No	317 (84.8%)	113 (88.3%)^a^	201 (82.7%)^a^	3 (100.0%)^a^	0.288	319 (84.6%)	174 (83.3%)	145 (86.3%)	0.414 (χ^2^)
	Yes	57 (15.2%)	15 (11.7%)^a^	42 (17.3%)^a^	0 (0%)^a^		58 (15.4%)	35 (16.7%)	23 (13.7%)	
Suicide plan – collision with a vehicle	No	350 (93.6%)	124 (96.9%)^a^	223 (91.8%)^a^	3 (100.0%)^a^	0.157	353 (93.6%)	199 (95.2%)	154 (91.7%)	0.161 (χ^2^)
	Yes	24 (6.4%)	4 (3.1%)^a^	20 (8.2%)^a^	0 (0%)^a^		24 (6.4%)	10 (4.8%)	14 (8.3%)	
Suicide plan – drug overdose	No	336 (89.8%)	109 (85.2%)^a^	225 (92.6%)^b^	2 (66.7%)^a, b^	0.025	339 (89.9%)	192 (91.9%)	147 (87.5%)	0.162 (χ^2^)
	Yes	38 (10.2%)	19 (14.8%)^a^	18 (7.4%)^b^	1 (33.3%)^a, b^		38 (10.1%)	17 (8.1%)	21 (12.5%)	
Suicide plan – animal attacks	No	373 (99.7%)	128 (100.0%)^a^	242 (99.6%)^a^	3 (100.0%)^a^	>0.999	376 (99.7%)	208 (99.5%)	168 (100.0%)	>0.999
	Yes	1 (0.3%)	0 (0%)^a^	1 (0.4%)^a^	0 (0%)^a^		1 (0.3%)	1 (0.5%)	0 (0%)	
Suicide plan – suffocation by carbon monoxide	No	370 (98.9%)	128 (100.0%)^a^	240 (98.8%)^a^	2 (66.7%)^b^	0.018	373 (98.9%)	205 (98.1%)	168 (100.0%)	0.132
	Yes	4 (1.1%)	0 (0%)^a^	3 (1.2%)^a^	1 (33.3%)^b^		4 (1.1%)	4 (1.9%)	0 (0%)	
Suicide plan – self-immolation (fire)	No	371 (99.2%)	128 (100.0%)^a^	240 (98.8%)^a^	3 (100.0%)^a^	0.565	374 (99.2%)	207 (99.0%)	167 (99.4%)	>0.999
	Yes	3 (0.8%)	0 (0%)^a^	3 (1.2%)^a^	0 (0%)^a^		3 (0.8%)	2 (1.0%)	1 (0.6%)	
Suicide plan – firearm	No	373 (99.7%)	128 (100.0%)^a^	242 (99.6%)^a^	3 (100.0%)^a^	>0.999	376 (99.7%)	208 (99.5%)	168 (100.0%)	>0.999
	Yes	1 (0.3%)	0 (0%)^a^	1 (0.4%)^a^	0 (0%)^a^		1 (0.3%)	1 (0.5%)	0 (0%)	
Suicide plan – electrocution	No	372 (99.5%)	128 (100.0%)^a^	241 (99.2%)^a^	3 (100.0%)^a^	0.554	375 (99.5%)	209 (100.0%)	166 (98.8%)	0.198
	Yes	2 (0.5%)	0 (0%)^a^	2 (0.8%)^a^	0 (0%)^a^		2 (0.5%)	0 (0%)	2 (1.2%)	
Suicide plan – suicide by cop	No	373 (99.7%)	128 (100.0%)^a^	242 (99.6%)^a^	3 (100.0%)^a^	>0.999	376 (99.7%)	208 (99.5%)	168 (100.0%)	>0.999
	Yes	1 (0.3%)	0 (0%)^a^	1 (0.4%)^a^	0 (0%)^a^		1 (0.3%)	1 (0.5%)	0 (0%)	
Suicide attempt – drinking chemicals/poison	No	344 (88.2%)	128 (87.1%)^a^	214 (88.8%)^a^	2 (100.0%)^a^	0.712	351 (88.4%)	202 (89.8%)	149 (86.6%)	0.331 (χ^2^)
	Yes	46 (11.8%)	19 (12.9%)^a^	27 (11.2%)^a^	0 (0%)^a^		46 (11.6%)	23 (10.2%)	23 (13.4%)	
Suicide attempt – jumping	No	371 (95.1%)	142 (96.6%)^a^	227 (94.2%)^a^	2 (100.0%)^a^	0.403	378 (95.2%)	216 (96.0%)	162 (94.2%)	0.401 (χ^2^)
	Yes	19 (4.9%)	5 (3.4%)^a^	14 (5.8%)^a^	0 (0%)^a^		19 (4.8%)	9 (4.0%)	10 (5.8%)	
Suicide attempt – drowning	No	382 (97.9%)	146 (99.3%)^a^	234 (97.1%)^a^	2 (100.0%)^a^	0.298	389 (98.0%)	221 (98.2%)	168 (97.7%)	0.731
	Yes	8 (2.1%)	1 (0.7%)^a^	7 (2.9%)^a^	0 (0%)^a^		8 (2.0%)	4 (1.8%)	4 (2.3%)	
Suicide attempt – cutting and stabbing	No	359 (92.1%)	132 (89.8%)^a^	225 (93.4%)^a^	2 (100.0%)^a^	0.363	365 (91.9%)	208 (92.4%)	157 (91.3%)	0.673 (χ^2^)
	Yes	31 (7.9%)	15 (10.2%)^a^	16 (6.6%)^a^	0 (0%)^a^		32 (8.1%)	17 (7.6%)	15 (8.7%)	
Suicide attempt – hanging	No	345 (88.5%)	136 (92.5%)^a^	207 (85.9%)^b^	2 (100.0%)^a, b^	0.104	351 (88.4%)	198 (88.0%)	153 (89.0%)	0.769 (χ^2^)
	Yes	45 (11.5%)	11 (7.5%)^a^	34 (14.1%)^b^	0 (0%)^a, b^		46 (11.6%)	27 (12.0%)	19 (11.0%)	
Suicide attempt – collision with a vehicle	No	379 (97.2%)	145 (98.6%)^a^	232 (96.3%)^a^	2 (100.0%)^a^	0.262	386 (97.2%)	222 (98.7%)	164 (95.3%)	0.063
	Yes	11 (2.8%)	2 (1.4%)^a^	9 (3.7%)^a^	0 (0%)^a^		11 (2.8%)	3 (1.3%)	8 (4.7%)	
Suicide attempt – drug overdose	No	367 (94.1%)	135 (91.8%)^a^	230 (95.4%)^a^	2 (100.0%)^a^	0.277	374 (94.2%)	216 (96.0%)	158 (91.9%)	0.080 (χ^2^)
	Yes	23 (5.9%)	12 (8.2%)^a^	11 (4.6%)^a^	0 (0%)^a^		23 (5.8%)	9 (4.0%)	14 (8.1%)	
Suicide attempt – animal attacks	No	390 (100.0%)	147 (100.0%)^a^	241 (100.0%)^a^	2 (100.0%)^a^	-	397 (100.0%)	225 (100.0%)	172 (100.0%)	–
	Yes	0 (0%)	0 (0%)	0 (0%)	0 (0%)		0 (0%)	0 (0%)	0 (0%)	
Suicide attempt – suffocation by carbon monoxide	No	389 (99.7%)	147 (100.0%)^a^	240 (99.6%)^a^	2 (100.0%)^a^	>0.999	396 (99.7%)	224 (99.6%)	172 (100.0%)	>0.999
	Yes	1 (0.3%)	0 (0%)^a^	1 (0.4%)^a^	0 (0%)^a^		1 (0.3%)	1 (0.4%)	0 (0%)	
Suicide attempt – self-immolation (fire)	No	389 (99.7%)	147 (100.0%)^a^	240 (99.6%)^a^	2 (100.0%)^a^	>0.999	396 (99.7%)	225 (100.0%)	171 (99.4%)	0.433
	Yes	1 (0.3%)	0 (0%)^a^	1 (0.4%)^a^	0 (0%)^a^		1 (0.3%)	0 (0%)	1 (0.6%)	
Suicide attempt – firearm	No	389 (99.7%)	147 (100.0%)^a^	240 (99.6%)^a^	2 (100.0%)^a^	>0.999	395 (99.5%)	224 (99.6%)	171 (99.4%)	>0.999
	Yes	1 (0.3%)	0 (0%)^a^	1 (0.4%)^a^	0 (0%)^a^		2 (0.5%)	1 (0.4%)	1 (0.6%)	
Suicide attempt – electrocution	No	389 (99.7%)	147 (100.0%)^a^	240 (99.6%)^a^	2 (100.0%)^a^	>0.999	396 (99.7%)	224 (99.6%)	172 (100.0%)	>0.999
	Yes	1 (0.3%)	0 (0%)^a^	1 (0.4%)^a^	0 (0%)^a^		1 (0.3%)	1 (0.4%)	0 (0%)	
Suicide attempt – suicide by cop	No	389 (99.7%)	147 (100.0%)^a^	240 (99.6%)^a^	2 (100.0%)^a^	>0.999	396 (99.7%)	225 (100.0%)	171 (99.4%)	0.433
	Yes	1 (0.3%)	0 (0%)^a^	1 (0.4%)^a^	0 (0%)^a^		1 (0.3%)	0 (0%)	1 (0.6%)	

*P*-values are from Fisher's exact test unless otherwise specified. Superscript letters (a, b) denote a subset of gender categories whose column proportions did not differ significantly from each other at the 0.05 level.

Suicide attempt was associated with having a jumping suicide plan (χ^2^ = 6.81; *P* = 0.009) or a drug overdose suicide plan (χ^2^ = 4.01; *P* = 0.045). In the univariate analysis, suicide attempt was positively associated with a jumping suicide plan. Participants with a jumping suicide plan had 60% lower odds of attempting suicide than those who did not have a jumping suicide plan. The multivariate analysis revealed that suicide attempts were significantly predicted by participants having a jumping suicide plan. See Table 4 for details of suicide plan methods associated with attempts.

**Table 4:** Suicide plan methods associated with attempt

Suicide plan	Category	Overall (*N* = 2652)	Total (*N* = 2534)	Suicide attempt		*P*-value	Univariate logistic regression model		Multivariate logistic regression model	
				No	Yes		COR (95% CI)	*P*-value	AOR (95% CI)	*P*-value
Drinking chemicals/poison	No	306 (81.2%)	306 (81.2%)	100 (32.7%)	206 (67.3%)	0.856 (χ^2^)	Ref.		Ref.	
	Yes	71 (18.8%)	71 (18.8%)	24 (33.8%)	47 (66.2%)		0.95 (0.55–1.66)	0.856	0.94 (0.53–1.69)	0.825
Jumping	No	343 (91.0%)	343 (91.0%)	106 (30.9%)	237 (69.1%)	**0.009** (χ^2^)	Ref.		Ref.	
	Yes	34 (9.0%)	34 (9.0%)	18 (52.9%)	16 (47.1%)		**0.40 (0.19–0.81)**	**0.011**	**0.41 (0.19–0.85)**	**0.016**
Drowning	No	362 (96.0%)	362 (96.0%)	118 (32.6%)	244 (67.4%)	0.580	Ref.		Ref.	
	Yes	15 (4.0%)	15 (4.0%)	6 (40%)	9 (60%)		0.73 (0.26–2.21)	0.551	0.90 (0.30–3.05)	0.857
Cutting and stabbing	No	343 (91.0%)	343 (91.0%)	114 (33.2%)	229 (66.8%)	0.651 (χ^2^)	Ref.		Ref.	
	Yes	34 (9.0%)	34 (9.0%)	10 (29.4%)	24 (70.6%)		1.19 (0.57–2.70)	0.651	1.24 (0.58–2.86)	0.588
Hanging	No	319 (84.6%)	319 (84.6%)	105 (32.9%)	214 (67.1%)	0.981 (χ^2^)	Ref.		Ref.	
	Yes	58 (15.4%)	58 (15.4%)	19 (32.8%)	39 (67.2%)		1.01 (0.56–1.86)	0.981	0.96 (0.52–1.81)	0.897
Collision with a vehicle	No	353 (93.6%)	353 (93.6%)	115 (32.6%)	238 (67.4%)	0.656 (χ^2^)	Ref.		Ref.	
	Yes	24 (6.4%)	24 (6.4%)	9 (37.5%)	15 (62.5%)		0.81 (0.35–1.97)	0.620	0.83 (0.35–2.07)	0.677
Drug overdose	No	339 (89.9%)	339 (89.9%)	117 (34.5%)	222 (65.5%)	**0.045** (χ^2^)	Ref.		Ref.	
	Yes	38 (10.1%)	38 (10.1%)	7 (18.4%)	31 (81.6%)		2.33 (0.99–5.46)	0.051	2.13 (0.94–5.47)	0.087
Animal attacks	No	376 (99.7%)	376 (99.7%)	123 (32.7%)	253 (67.3%)	0.329	Ref.		Ref.	
	Yes	1 (0.3%)	1 (0.3%)	1 (100%)	0 (0%)		0.00 (0.00–Inf.)	0.979	0.00 (0.00–Inf.)	0.991
Suffocation by carbon monoxide	No	373 (98.9%)	373 (98.9%)	122 (32.7%)	251 (67.3%)	0.601	Ref.		Ref.	
	Yes	4 (1.1%)	4 (1.1%)	2 (50%)	2 (50%)		0.49 (0.06–4.09)	0.473	0.93 (0.09–20.2)	0.951
Self-immolation (fire)	No	374 (99.2%)	374 (99.2%)	123 (32.9%)	251 (67.1%)	>0.999	Ref.		Ref.	
	Yes	3 (0.8%)	3 (0.8%)	1 (33.3%)	2 (66.7%)		0.98 (0.09–21.2)	0.987	0.93 (0.09–20.2)	0.951
Firearm	No	376 (99.7%)	376 (99.7%)	123 (32.7%)	253 (67.3%)	0.329	Ref.		Ref.	
	Yes	1 (0.3%)	1 (0.3%)	1 (100%)	0 (0%)		0.00 (0.00–Inf.)	0.979	0.00 (0.00–Inf.)	0.991
Electrocution	No	375 (99.5%)	375 (99.5%)	123 (32.8%)	252 (67.2%)	0.550	Ref.		Ref.	
	Yes	2 (0.5%)	2 (0.5%)	1 (50%)	1 (50%)		0.49 (0.02–12.4)	0.613	0.73 (0.03–19.8)	0.828
Suicide by cop	No	376 (99.7%)	376 (99.7%)	124 (33%)	252 (67%)	>0.999	Ref.		Ref.	
	Yes	1 (0.3%)	1 (0.27%)	0 (0%)	1 (100%)		383 433 (0.00–Inf.)	0.981	2 667 911 (0.00–Inf.)	0.992

COR, crude odds ratio; AOR, adjusted odds ratio; Ref., reference category; Inf., positive large number.

*P*-values are from Fisher's exact test unless otherwise specified. Bold indicates significant results.

### Sociodemographic associations of suicidal thoughts, plans and attempts

Suicidal thoughts were significantly associated with age (*P* = <0.001), school form (χ^2^ = 20.46; *P* = <0.001) and location of school (χ^2^ = 12.61; *P* = <0.001). Those with ‘other’ gender had five times greater odds of suicidal thoughts than males. For a 1 year increase in age, there was a 20% increase in the odds of suicidal thoughts. Participants in forms 3 and 4 (the third and final years in high school, respectively) had greater odds of thinking about suicide compared with those in form 1 (the first year in high school). Location of school was also a key determinant of suicidal thoughts, with respondents in urban areas more likely to think about suicide than those in rural areas. Suicidal thoughts were significantly predicted by female gender and older age.

Suicide plans were significantly associated with age (*P* = <0.001), school form/class (χ^2^ = 16.72; *P* = <0.001) and location of school (χ^2^ = 6.65; *P* = 0.05). For a 1 year increase in age, there was an 18% increase in the odds of having a suicide plan. Participants in forms 3 and 4 had greater odds of suicide plans compared with those in form 1. Location of school (rural or urban) was also a key determinant of suicide plans, with respondents in urban areas more likely to plan suicide than those in rural areas. Suicide plans were significantly predicted by ‘other’ gender.

Suicide attempts were significantly associated with gender (χ^2^ = 5.69; *P* = 0.046), age (*P* = 0.002), school form (χ^2^ = 9.17; *P* = 0.027) and location of school (χ^2^ = 4.44; *P* = 0.035). Females had greater odds of attempting suicide than males. For a one-unit increase in age, there was a 15% increase in the odds of a suicide attempt. Participants in form 4 had greater odds of attempting suicide compared with those in form 1. The location of the school was also a key determinant of suicide attempts, with respondents in urban areas more likely to attempt suicide than those in rural areas. In the multivariate analysis, suicide attempts were significantly predicted by female gender and older age. See Table 5 for details of sociodemographic associations of thoughts, plans and attempts.

**Table 5:** Sociodemographic associations of thoughts, plans and attempts

Sociodemographic factor	Category	Overall (*N* = 2652)	Total (*N* = 2534)	Suicidal thoughts	Suicide plan	Suicide attempt	Univariable logistic regression			Multivariable logistic regression		
				(*n* = 680)	(*n* = 377)	(*n* = 397)	Suicide thought	Suicide plan	Suicide attempt	Suicide thought	Suicide plan	Suicide attempt
							COR (95% CI)	COR (95% CI)	COR (95% CI)	AOR (95% CI)	AOR (95% CI)	AOR (95% CI)
Gender	Female	862 (33.2%)	832 (33.4%)	232 (27.9%)	128 (15.4%)	147 (17.7%)	1.09 (0.90–1.31)	1.05 (0.83–1.33)	1.26 (1.00–1.57)*	1.33 (1.07–1.66)*	1.30 (0.99–1.71)	1.50 (1.15–1.96)**
	Male	1728 (66.6%)	1651 (66.3%)	433 (26.2%)	243 (14.7%)	241 (14.6%)	Ref.	Ref.	Ref.	Ref.	Ref.	Ref.
	Other	6 (0.2%)	6 (0.2%)	4 (66.7%)	3 (50%)	2 (33.3%)	5.63 (1.09–40.7)*	5.79 (1.07–31.5)*	2.93 (0.40–15.1)	5.61 (0.51–125)	12.6 (1.16–279)*	0.00 (0.00–1 081 642 197 143)
Age (Years)	Mean (s.d.)	16.13 (1.38)	16.13 (1.39)	16.38 (1.38)	16.40 (1.37)	16.36 (1.39)	1.20 (1.11–1.29)***	1.18 (1.08–1.29)***	1.15 (1.05–1.26)**	1.15 (1.03–1.29)*	1.12 (0.98–1.28)	1.19 (1.04–1.36)**
Form	1	869 (33.5%)	831 (33.4%)	189 (22.7%)	96 (11.6%)	112 (13.5%)	Ref.	Ref.	Ref.	Ref.	Ref.	Ref.
	2	646 (24.9%)	615 (24.7%)	166 (27%)	87 (14.1%)	103 (16.7%)	1.26 (0.99–1.60)	1.26 (0.92–1.72)	1.29 (0.97–1.73)	1.18 (0.87–1.59)	1.18 (0.81–1.73)	1.20 (0.83-1.72)
	3	729 (28.1%)	704 (28.3%)	193 (27.4%)	121 (17.2%)	107 (15.2%)	1.28 (1.02–1.62)*	1.59 (1.19–2.12)**	1.15 (0.86–1.53)	1.15 (0.82–1.62)	1.39 (0.91–2.12)	0.92 (0.60–1.40)
	4	352 (13.6%)	340 (13.7%)	121 (35.6%)	67 (19.7%)	69 (20.3%)	1.88 (1.42–2.47)***	1.88 (1.33–2.64)***	1.63 (1.17–2.27)**	1.33 (0.84–2.12)	1.26 (0.71–2.24)	1.00 (0.57–1.76)
Location of school	Rural	1627 (61.3%)	1556 (61.4%)	379 (24.4%)	209 (13.4%)	225 (14.5%)	Ref.	Ref.	Ref.	Ref.	Ref.	Ref.
	Urban	1025 (38.7%)	978 (38.6%)	301 (30.8%)	168 (17.2%)	172 (17.6%)	1.38 (1.15–1.65)***	1.34 (1.07–1.67)*	1.26 (1.02–1.57)*	1.20 (0.97–1.48)	1.17 (0.89–1.52)	1.11 (0.85–1.45)

COR, crude odds ratio; AOR, adjusted odds ratio; Ref., reference category.

*P < 0.05, **P < 0.01, ***P < 0.001.

### Combinations of suicidality

Table 6 summarises the different combinations and overlaps of suicidal thoughts, plans and attempts and their associations with sociodemographic variables. Table 1 and Fig. 1 summarise the different patterns found in suicide attempts. These patterns were:

- suicidal thoughts and plans associated with attempts;
- suicidal thoughts and attempts not associated with plans;
- attempts not associated with thoughts or plans.

**Table 6:** Associations of different suicidality combinations with sociodemographic factors

Sociodemographic factor	Category	Total (*N* = 2534)	Thoughts + plans + attempts	Thoughts + no plans + attempts	No thoughts + no plans + attempts	Univariable logistic regression			Multivariable logistic regression		
			(*n* = 253)	(*n* = 96)	(*n* = 48)	Suicide – thoughts + plans + attempts	Suicide – thoughts + no plans + attempts	Suicide – no thoughts + no plans + attempts	Suicide – thoughts + plans + attempts	Suicide – thoughts + no plans + attempts	Suicide – no thoughts + no plans + attempts
						COR (95% CI)	COR (95% CI)	COR (95% CI)	AOR (95% CI)	AOR (95% CI)	AOR (95% CI)
Gender	Female	832 (33.4%)	92 (11.1%)	37 (4.4%)	18 (2.2%)	1.18 (0.90–1.55)	1.38 (0.89–2.10)	1.19 (0.65–2.14)	1.45 (1.05–1.99)*	1.82 (1.07–3.05)*	0.90 (0.41–1.84)
	Male	1651 (66.3%)	157 (9.5%)	54 (3.3%)	30 (1.8%)	Ref.	Ref.	Ref.	Ref.	Ref.	Ref.
	Other	6 (0.24%)	1 (16.7%)	1 (16.7%)	0 (0%)	1.90 (0.10–11.9)	5.91 (0.31–37.5)	0.00 (0.00–49 006 468 274 241 781 760)	0.00 (0.00–121 859 588 488 368 247 282 420)	0.00 (0.00–3 190 494 327 841 758 989 668 800 440 004 402 848 484)	0.00 (0.00–37 270 160 883 709 969 720 624 406 022 660 840 044 884)
Age (Years)	Mean (s.d.)	16.13 (1.39)	16.45 (1.35)	16.55 (1.47)	15.47 (1.16)	1.20 (1.08–1.33)***	1.24 (1.05–1.47)*	0.68 (0.52–0.89)**	1.16 (0.99-1.36)	1.54 (1.20–1.94)***	0.68 (0.45–1.00)
Form	1	831 (33.4%)	63 (7.6%)	30 (3.6%)	19 (2.3%)	Ref.	Ref.	Ref.	Ref.	Ref.	Ref.
	2	615 (24.7%)	59 (9.6%)	30 (4.9%)	14 (2.3%)	1.29 (0.89–1.88)	1.37 (0.81–2.30)	1.00 (0.49–1.99)	1.25 (0.80–1.97)	1.24 (0.63–2.50)	1.06 (0.41–2.60)
	3	704 (28.3%)	79 (11.2%)	18 (2.6%)	10 (1.4%)	1.54 (1.09–2.19)*	0.70 (0.38–1.26)	0.62 (0.27–1.31)	1.29 (0.78–2.14)	0.40 (0.16–0.96)*	1.05 (0.31–3.38)
	4	340 (13.7%)	49 (14.4%)	16 (4.7%)	4 (1.2%)	2.05 (1.38–3.05)***	1.32 (0.69–2.42)	0.51 (0.15–1.37)	1.37 (0.70–2.69)	0.45 (0.15–1.33)	1.00 (0.12–5.82)
Location of school	Rural	1556 (61.4%)	143 (9.2%)	51 (3.3%)	31 (2%)	Ref.	Ref.	Ref.	Ref.	Ref.	Ref.
	Urban	978 (38.6%)	110 (11.2%)	45 (4.6%)	17 (1.7%)	1.25 (0.96–1.63)	1.42 (0.94–2.14)	0.87 (0.47–1.56)	1.06 (0.77–1.45)	1.35 (0.79–2.27)	0.91 (0.42–1.86)

COR, crude odds ratio; AOR, adjusted odds ratio; Ref., reference category.

*P< 0.05, **P < 0.01, ***P < 0.001.

Gender, age, school form and location of school were associated with suicidal attempts.

## Discussion

We present the first study of the prevalence of suicidal thoughts, suicidal plans and suicidal attempts in Kenyan high school students. This was also the first Kenyan study to report on the methods conceived by students as part of their plans to end their life, as well as the different methods used in their suicide attempts. We report that although there is an assumed association of suicide attempts with plans and attempts, this is not always the case; suicide attempts were not always associated with thoughts and/or plans. However, the probability of having plans was doubled for those with thoughts, and those with plans had a much higher probability of making attempts. However, this is a cross-sectional situation. We had no information on the sequential progression over time in a given individual. This would be best addressed by both qualitative and longitudinal studies. However, our findings underline the importance of vigilance regarding any suicidal thoughts and, in particular, suicidal plans. As not all suicidal attempts are associated with thoughts or plans, vigilance should also be applied to the associations we report here. We have demonstrated that there are sociodemographic and environmental factors that are significantly associated with attempts and that also predict attempts. There are also environmental factors related to the availability of certain methods that could be manipulated to mitigate suicide.

### Response rate

The high response rate of 95.6% (*n* = 2534 out of 2652 participants) was not unique to this study. Similar rates have been consistently found in Kenyan studies, particularly those involving school-going children.^18,19^ There are several explanations, including our approach of explaining the nature of the study to schools and communities to achieve buy-in, and the willingness to participate in an activity that has the potential to improve mental health – specifically, the mental health of students – and in turn improve academic performance.

### Prevalence of thoughts, plans and attempts

The overall prevalence of 26.8% suicidal thoughts was well above rates reported for Western countries but within the range reported for other African countries, as referenced earlier in this paper. The disparity in prevalence between these regions could be due to socioeconomic factors.^1^ African countries often face greater economic instability, poverty and lack of access to basic resources such as healthcare, education and employment opportunities, which can contribute to higher levels of stress and hopelessness, increasing vulnerability to suicide. Furthermore, mental health services and resources are often limited in these countries, and the stigma surrounding mental illness may also prevent individuals from seeking help or accessing appropriate care even where services are available.^20,21^

However, the prevalence rates of 14.9% for plans and 15.7% for attempts were above those reported in the literature, as indicated earlier. These may reflect the prevailing situation, given the timing of the study with respect to events including the COVID-19 pandemic; media reports of war in Europe; an election year in Kenya, with threats of violence associated with past elections; and the consequences of climate change, presenting in Kenya with unusual extremes of temperature, droughts (failure of crops, death of livestock) often alternating with deadly floods.^22^ Even those students who did not have personal experience with these factors would have seen many reports in the media, potentially leading to vicarious trauma. In addition, the sample included students in the final year of high school, facing high-stakes exams and thus pressure to excel. Not all suicidal attempts were associated with thoughts and plans. This poses a challenge in predicting suicidal attempts, and there is thus a need for reliance on other associations of suicide attempts, as well as simple screening of mental health in schools.^23^

### Methods in suicidal plans and attempts

Methods were similar for plans and attempts, and they appeared to reflect availability and ease of access; this suggests that an important step toward preventing suicide may be to limit access to those methods. Our study found taking chemicals and poisons to be the leading method reported in plans and attempts in the Kenyan context, a finding similar to that of the WHO report referenced earlier. This finding is not surprising, because in Kenya these chemicals are widely available in homes and at agro-vet outlets for the control of vermin at homesteads and farms, and as herbicides and pesticides for agricultural activities such as the farming of tea, coffee and other crops and of livestock. These are major sources of income for families and the country. There is, therefore, a need for alternatives that are less injurious to human beings, coupled with regulations on availability and access and appropriate secure storage at home. This should be amplified by an awareness campaign targeting communities and families, especially those who use such poisons and chemicals for the economic activities specified above. The same general principles should be applied in relation to drug overdose (the third most common method), especially in relation to regulated access and availability, for prescribed drugs in particular, as well as involving caregivers at home in the oversight and safe custody of prescribed drugs at home. It is noteworthy that participants with a drug overdose suicide plan had two times greater odds of suicide attempt, reflecting easy access to these drugs.

It may be especially challenging to prevent suicides by hanging or cutting because of the wide range of options available at home and outside home. Raising caregiver awareness is paramount. To some extent, jumping from heights (buildings, bridges, etc.) can be mitigated by installing barriers on parapets, windows and other areas where people are likely to jump. It was noteworthy that in our study, having a jumping plan was a significant predictor of suicide attempts but in a negative direction; that is, participants with a jumping suicide plan had lower odds of attempting suicide than those who did not have a suicide plan. Worrisome, though, is the fact that methods traditionally associated with settings outside Kenya – cutting oneself, for example – are beginning to enter the scene in Kenya.

The fact that the students we surveyed had survived their attempts and could thus tell their stories may be a reflection of the mild severity of the attempts or the low lethality of the methods used. Overall, the findings suggest that exerting control over students’ environment at home, school and community may go a long way towards preventing youth suicide, and public and family awareness of methods could contribute to the prevention of suicide.

### Mitigating the association of suicidal attempts with thoughts and plans

First, our findings suggest very strongly the need to take suicidal thoughts and plans seriously and on their own merit. This is because those with suicidal thoughts have an increased prevalence of plans, and those with plans have an increased prevalence of attempts (with the caveat that this was a cross-sectional population study, and we had no information on sequential or different staring points for a given individual). Second, our findings suggest that focusing only on thoughts and/or plans as necessary precursors of attempts may be inadequate, as nearly one-third of attempts appeared to be unanticipated on the basis of thoughts and plans.

### Sociodemographic and environmental factors associated with suicidal attempts

We emphasise sociodemographic factors and other environmental factors because these are key to potential entry points for interventions; in particular, environmental factors that are amenable to manipulation as part of mitigation, for example, the availability of chemical and poisons, and the need for caregivers to be aware of various methods and hotspots in the community and at home. To this end, our findings confirm literature reports, including our own earlier findings,^1^ that older age (here reflected by increasing seniority of high school students) and urban rather than rural areas are associated with suicidality. Female gender and age are independently associated with suicidal attempts. In the Kenyan context, an important finding is that form 4 students, the most senior students in high school, are particularly vulnerable to suicide attempts. This final year is the most stressful as the students prepare for their National Examination, which will affect their future educational and career opportunities, i.e. whether or not they will qualify to continue their education by gaining admission to colleges and universities. Our study provides more information on environmental factors related to available methods; these environmental factors could be manipulated to mitigate suicidal attempts and suicide.

In summary, suicidal thoughts, plans and attempts are prevalent in Kenyan high school students. There are multiple methods with different frequencies of use, with taking chemicals and poisons being the leading method reported in plans and attempts. There are different starting points before suicidal attempts. Although the most common and well-documented starting point is from suicidal thoughts to plans to attempts, there are other starting points to attempts that do not include thoughts and plans. These other starting points may not lend themselves to detection at clinical and community levels except to the extent that various other associations can be identified, in particular, sociodemographic characteristics and environmental factors related to the availability of certain methods. There is, therefore, a need for reliance on the other various associations of suicide attempts. Being a senior-level student and going to a school in an urban area were associated with increased suicide attempts, whereas being in the final year of high school and female gender were independently associated with suicide attempts.

### Strengths and limitations of the study

This was a cross-sectional study; thus, no causalities were drawn. Neither could we determine the different starting points for a given individual. As we used a self-report survey, there was a likelihood of social desirability bias, where participants might have responded to questions in a way that they perceived to be socially acceptable or desirable, rather than providing honest or accurate answers. Although this study was conducted in groups meeting in classrooms, with students filling out the questionnaire at their own pace, the students were given the option not to answer any question they did not want to answer. We only worked with the responses that the students were willing to provide, so it is possible that our findings would have been different in some respects if we had had all the information that some students chose not to provide. This study may not represent Kenyan high school students generally, as it was conducted in only three counties, which do not fully account for sociocultural differences across Kenya. In addition, the very sad fact that only those whose suicide attempts were unsuccessful could participate in the study meant that we were not able to assess associations of lethal suicide attempts. The focus of this study was on the prevalence of thoughts, plans and attempts, methods and modifiable environmental factors. It did not explore the mental health aspects. A major strength is the high response rate, as discussed above.

### Recommendations

To understand the sequential progression from thoughts to plans to attempts we recommend a prospective study using mixed methods, i.e. qualitative and quantitative.

## Data Availability

Requests for the data may be sent to the corresponding author.
